# Placement of an antibiotic oral pack on the hard palate after primary cleft palatoplasty: a randomized controlled trial into the effect on fistula rates

**DOI:** 10.1007/s00784-017-2286-9

**Published:** 2017-11-30

**Authors:** Rajgopal R. Reddy, Srinivas Gosla Reddy, Bhavya Banala, Ewald M. Bronkhorst, Ann W. Kummer, Anne Marie Kuijpers-Jagtman, Stefaan J. Bergé

**Affiliations:** 1 Cranio-maxillofacial Surgery, G.S.R. Hospital, Institute of Cranio-Maxillofacial and Facial Plastic Surgery, Vinay Nagar Colony, Saidabad, Hyderabad, India; 2 Department of Dentistry, Radboud University Medical Center, Nijmegen, The Netherlands; 3 GSR Institute of Craniofacial Surgery, # 17-1-383/55, Vinay Nagar Colony, IS Sadan, Saidabad, Hyderabad, 500059 India; 4 Speech and Language Therapy, G.S.R. Hospital, Institute of Cranio-Maxillofacial and Facial Plastic Surgery, Vinay Nagar Colony, Saidabad, Hyderabad, India; 5 Department of Cariology and Preventive Dentistry, Radboud University Medical Center, Nijmegen, The Netherlands; 6 Division of Speech-Language Pathology, Cincinnati Children’s Hospital Medical Center, University of Cincinnati College of Medicine, Cincinnati, OH USA; 7 Department of Orthodontics and Craniofacial Biology, Radboud University Medical Center, Nijmegen, The Netherlands; 8 Department of Cranio-maxillofacial Surgery, Radboud University Medical Center, Nijmegen, The Netherlands

**Keywords:** Cleft palate, Surgical procedures, operative, Oral fistula, Treatment outcome

## Abstract

**Objective:**

The objective of this study is to determine whether placement of an antibiotic oral pack on the hard palate reduces fistula rates after primary cleft palatoplasty.

**Subjects and methods:**

This study was a parallel blocked randomized controlled trial. The study consisted of two groups of 100 patients each with non-syndromic unilateral complete cleft lip, alveolus, and hard and soft palate that underwent primary palatoplasty. Group A had an oral pack placed on the hard palate for 5 days postoperatively while group B did not. Occurrence of fistulae between both groups was tested using odds ratios (OR).

**Results:**

In 2% of the patients in group A, a fistula was found 6 months after palatal surgery. In contrast, in 21% of the patients in group B, a palatal fistula could be confirmed. The fistula occurrence in group A was statistically significantly lower than that in group B (OR = 0.0768, CI = [0.02 … 0.34], *p* < 0.001).

**Conclusion:**

The findings of this study provide evidence that the rate of fistula formation after primary palatoplasty is significantly reduced if a pack soaked with antibiotic cream is placed on the palate postoperatively for 5 days.

**Clinical relevance:**

The use of an antibiotic pack after cleft palate repair can be recommended to prevent occurrence of oronasal fistulae.

## Introduction

Primary closure of a cleft palate should result in an intact palate with separation of the oral and nasal cavity [1]. Breakdown of the primary cleft palate repair causes oronasal fistulas with consequent dysfunction. Such fistulas are reported to occur between 0 and 77.8% of patients after primary palatoplasty [2]. Though the breakdown of a primary repaired cleft palate could be due to a number of reasons, localized infection may be a significant cause.

Infection of any open post-surgical site is a known phenomenon, especially if the site is open to food particles. Furthermore, mechanical trauma to the hard palate after palatoplasty could be caused by the patient putting his/her fingers in the mouth, eating food that is not soft, or being bottle-fed with the feeding bottle nipple resting on the hard palate. Placing an oral pack made out of a folded piece of sterile gauze soaked in antibiotic cream on the hard palate for 5 days postoperatively could address any injury to the healing tissue caused by localized infection or mechanical trauma. The aim of this study was to investigate whether placement of an antibiotic oral pack on the hard palate reduces fistula rates after primary cleft palatoplasty.

## Materials and methods

### Trial design

This study was conducted to ascertain whether the placement of a postoperative oral pack reduces fistula rates after primary repair of the cleft palate. The study design was a parallel blocked randomized controlled trial. As this is a surgical trial, the surgeon and patients could not be blinded for the treatment.

This study was conducted from June 1, 2012 to August 31, 2013, at the GSR Institute of Craniofacial Surgery, Hyderabad, India, which is a high-volume cleft center where 1400 cleft surgeries are performed every year. The local Ethical Committee approved the research protocol (ETH/GSRICFS/2011/DEC 2) based on the guidelines declared by the Government of India. All participants and parents, if the participants were minors, were informed about the study and signed a written informed consent. Reporting of the trial in this paper follows the CONSORT (Consolidated Standards of Reporting Trials) statement [3].

### Eligibility and randomization

Inclusion criteria for this study were non-syndromic complete unilateral cleft lip and palate with a previously repaired cleft lip; palate repair was planned at the age of 12 months. Exclusion criteria were bilateral cleft lip and palate, isolated cleft palate, patients older than 13 months of age, and patients with associated syndromic conditions.

To detect a reduction of fistula rates with a placement of a palatal pack after primary palatoplasty, which we estimated to reduce by 15% with a two-sided 5% significance level and a power of 80%, a sample size of 200 was necessary. The intake period was anticipated to be 15 months to recruit the required number of patients.

Patients who fulfilled the eligibility criteria were randomly assigned to either group A or B. The randomization sequence was generated by a computer program (Sealedenvelope™, Sealed Envelope Ltd., London, UK) using blocked randomization in block sizes of 20 in each block. Within each block, participants were randomly assigned numbers by a computerized program to one of the two treatment groups. The randomization was performed by one speech therapist (BB). The surgeon was blinded to the randomization process. After assigning the treatment method, the patients’ parents were informed of the treatment plan by the speech therapist (BB). If the parents did not agree to the treatment plan assigned randomly to their child, the patient was excluded from the study and the number was assigned to the next patient on the list.

### Interventions

**Fig. 1:**
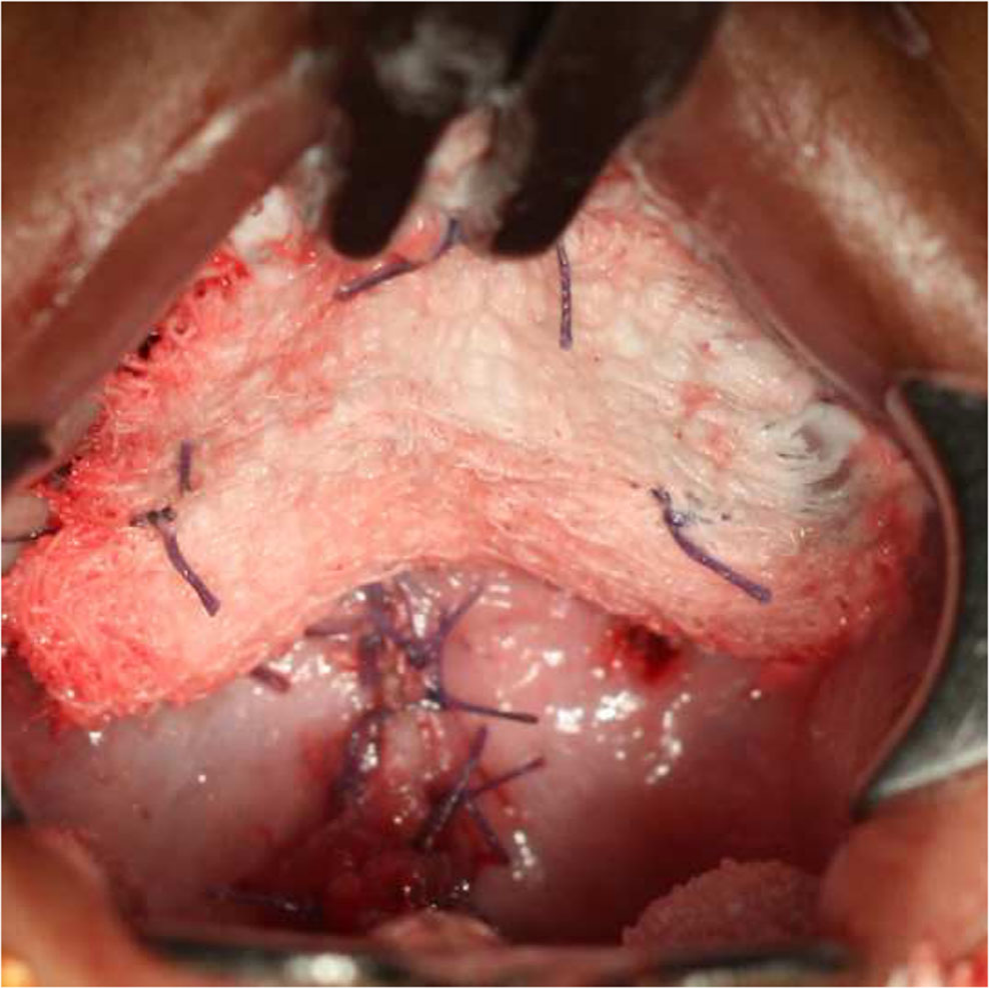
Palatal pack placed on the hard palate

One surgeon (RRR) performed palatal surgery on all patients in groups A and B. The Bardach two-flap technique with optimal muscle dissection was the surgery of choice in both groups. The patients in group A (*n* = 100) received an oral pack made of sterile cotton gauze soaked in framycetin sulfate antibiotic cream (Soframycin Skin Cream, Sanofi India Limited, India) for 5 days postoperatively (Fig. 1). This pack was sutured in such a way that it was closely adhering to the hard palate. The patients of group B (*n* = 100) had no pack placed postoperatively. In patients where the pack was placed, it was removed after 5 days. All patients in groups A and B were given intravenous and oral antibiotics, conforming to the hospitals’ pediatric surgical protocol, i.e., two doses of intravenous injection cefotaxime 25 mg/kg body weight 12th hourly for the first 24 h postoperatively. This is followed by oral suspension amoxicillin and clavulanic acid 25 mg/kg body weight 12th hourly and syrup metronidazole 20 mg/kg body weight 8th hourly. These medications are given for 5 days. Postoperative feeding was done orally for all patients included in this study.

### Outcome

Patients in groups A and B were recalled after 6 months to clinically examine them for presence of fistulae. A single examiner (RRR) performed the examination to elicit the presence or absence of fistula. The examiner was blinded to whether the patient had a pack placed postoperatively or not. Fistula occurrence was tested visually as the first stage. If there was no visual sign of a fistula, history of nasal regurgitation was elicited. If the patient and/or parent gave a history of nasal regurgitation, a blunt periodontal probe was used to confirm a fistula in the hard palate.

### Statistical methods

The statistical analysis was performed with SPSS version 22 (Chicago, IL, USA). Occurrence of fistulae in the study was tested using odds ratios.

## Results

**Fig. 2:**
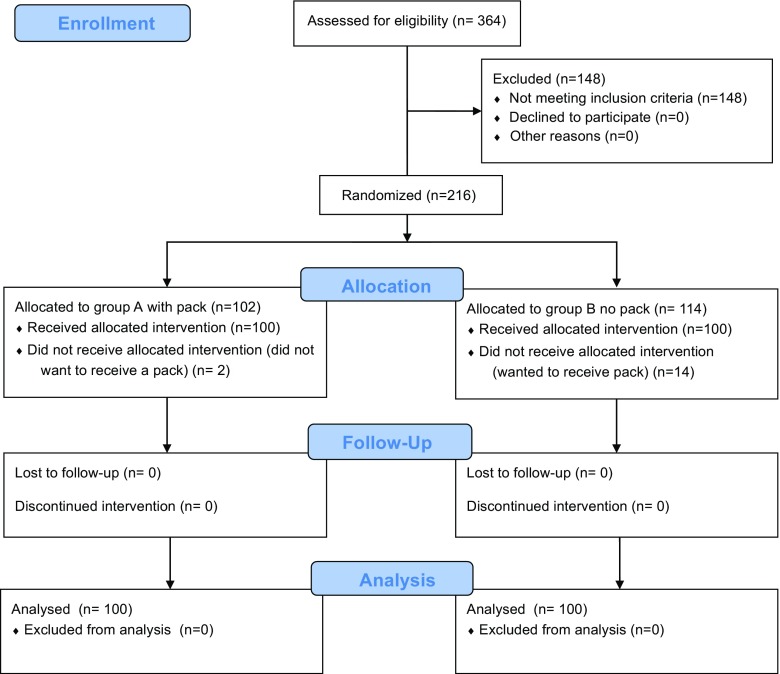
Flow diagram detailing the workflow through the trial

The flow of participants through each stage of the study is detailed in Fig. 2. All patients in groups A and B were operated between 12 and 13 months of age.

Of the patients in group A (with the oral pack), only 2% showed a fistula 6 months after palatal surgery. In contrast, in 21% of the patients in group B (without the oral pack), a palatal fistula could be confirmed. All the fistulae that were observed were present in the hard palate region. There were no fistulae present at the junction of the hard and soft palate. There was no instance of wound dehiscence in the soft palate region.

The fistula occurrence in group A was statistically significantly lower than that in group B (OR = 0.0768, CI = [0.02 … 0.34], *p* < 0.001) (Table 1).

**Table 1:** Odds ratio of fistula presence after primary palatoplasty with (group A) and without (group B) oral pack

Primary palatoplasty			
		Group A (*n* = 100) with pack	Group B (*n* = 100) no pack
Fistula	Yes	2	21
	No	98	79
	Total	100	100
	Odds ratio	0.0768	
	95% CI	[0.02 … 0.34]	
	*p*	< 0.001	

The fistula occurrence in group A was statistically significantly lower than that in group B

## Discussion

Effective palatal fistula management has to be practiced by cleft teams to ensure that occurrence is minimized. Nevertheless, the incidence of fistula occurrence after primary palatoplasty in patients with palatal clefts has been reported to range between 0 and 77.8% [2]. Based on a systematic review on incidence of palatal fistulae after primary palatoplasty performed by Hardwicke et al. in 2014, fistula rates in 44 studies included in the review ranged between 0 and 35% [4]. Five studies included in the systematic review showed no postoperative fistula [2, 5–8], while two studies reported a high fistula rate of 34 and 35%, respectively [9, 10].

Several studies have searched for an association between the severity of the palatal cleft and the rate of fistula formation [1, 11–13]. Some authors have attempted to isolate factors that would cause fistulae. The most common ones include tension of palatal soft tissue after palatal repair, upper respiratory infection, postoperative hemorrhage, failure of a multilayer closure, and cleft severity [1, 11]. However, none of the studies conclusively proved that the severity of the cleft has a clear association with fistula occurrence. There are no studies that have attempted to correlate the formation of fistulae with factors such as localized infection or mechanical trauma. The present study was performed to test a possible reduction of fistula rates by placing a temporary barrier between the hard palate and the oral environment to reduce mechanical trauma and by the use of an antibiotic cream to reduce infections. The antibiotic pack was kept in place for 5 days at which stage the proliferative phase leads to the maturation phase of healing by primary intention [14].

Various studies have used palatal splints, bandages, and other devices in order to reduce the occurrence of palatal fistulae. The most common appendage used is acellular dermal grafting [15–20]. Another possibility to protect the hard palate after closure is an acrylic splint or a celluloid acetone dressing [21, 22]. We preferred the antibiotic cream-soaked sterile gauze pack to other barriers like acellular dermal matrices or acrylic splints for a number of reasons. An antibiotic-soaked sterile gauze pack is readily available at the time of surgery and it is cost-effective; it does not need to be manipulated into a shape and once placed, it takes the natural shape of the palate due to the pressure exerted by the tongue. Acellular dermal matrix and iodoform gauze was not used due to their higher costs and difficulty to procure in India. Acrylic splints were not used due to the time taken for preparing a splint and the possibility of an adverse reaction of the palatal mucosa to acrylic.

This study was a parallel blocked randomized controlled trial. The patients were divided into two groups that received an antibiotic-soaked gauze pack (group A) and did not have any pack placed postoperatively (group B). In this study design, we cannot exclude that the antibiotic cream in the pack had a positive influence. As we found in the present study that the rate of fistula formation after primary palatoplasty is significantly reduced if a pack soaked with antibiotic cream is placed on the palate as compared to no pack, we could perform another study in the future comparing an oral pack with and without antibiotic cream. Before performing such a study, we need to know if food debris adherence to the pack could be a focus of infection for an open post-surgical wound in the oral cavity. Though we did not find any literature to correlate such a presumption, we assumed that a pack made of gauze without antibiotic cream could be detrimental to the patient. This study also excluded patients that did not agree with the treatment plan randomly assigned to them. This was done in contravention of the intention to treat principle of randomized controlled trials. However, though the patients were excluded from the study, the treatment plan of the patients was completed as requested by them.

Different surgical techniques have been used for primary repair of the cleft in the hard palates like the Bardach two-flap, von Langenbeck, and single-layer mucoperiosteal flaps [23, 24]. Similarly, different techniques like local mucoperiosteal flaps, turnover flaps from the palate, tongue flaps, pharyngeal flaps, buccal myomucosal flaps, facial artery musculo-mucosal flaps, free grafts of bone, cartilage, or dermal fat, free tissue transfer for large or recalcitrant fistulae, and acellular dermal matrix [25–37] as well as tissue engineering techniques [38] have been used to treat recurring fistulae. In the present study, local mucoperiosteal flaps were used in both groups (A and B) to repair the hard palate. To further standardize this study, all patients were treated using the Bardach two-flap technique. This ensured that a homogeneous group of patients treated by the same technique and by the same surgeon was studied for outcomes. The odds ratio of fistula formation after primary palatoplasty in children who did not have a pack placed increased statistically significantly. This means placing a pack postoperatively for patients undergoing primary palatoplasty was beneficial in context of oronasal fistulae in the hard palate. Which type of palatal pack is to be preferred in terms of fistula rate, patient comfort, and cost-effectiveness needs to be investigated further.

## Conclusion

The results of this study provide evidence that the rate of fistula formation after primary palatoplasty is significantly reduced if a pack soaked with antibiotic cream is placed on the palate postoperatively for 5 days.
